# USI: a fast and accurate approach for conceptual document annotation

**DOI:** 10.1186/s12859-015-0513-4

**Published:** 2015-03-14

**Authors:** Nicolas Fiorini, Sylvie Ranwez, Jacky Montmain, Vincent Ranwez

**Affiliations:** 1 LGI2P research center from the Ecole des mines d’Alès, Site de Nîmes, Parc scientifique G. Besse, Nîmes cedex 1, 30035 France; 2 UMR AGAP, Montpellier SupAgro/CIRAD/INRA, 2 place Pierre Viala, Montpellier Cedex 1, 34060 France

**Keywords:** Semantic annotations, Algorithms, Complexity, Benchmarking

## Abstract

**Background:**

Semantic approaches such as concept-based information retrieval rely on a corpus in which resources are indexed by concepts belonging to a domain ontology. In order to keep such applications up-to-date, new entities need to be frequently annotated to enrich the corpus. However, this task is time-consuming and requires a high-level of expertise in both the domain and the related ontology. Different strategies have thus been proposed to ease this indexing process, each one taking advantage from the features of the document.

**Results:**

In this paper we present USI (User-oriented Semantic Indexer), a fast and intuitive method for indexing tasks. We introduce a solution to suggest a conceptual annotation for new entities based on related already indexed documents. Our results, compared to those obtained by previous authors using the MeSH thesaurus and a dataset of biomedical papers, show that the method surpasses text-specific methods in terms of both quality and speed. Evaluations are done via usual metrics and semantic similarity.

**Conclusions:**

By only relying on neighbor documents, the User-oriented Semantic Indexer does not need a representative learning set. Yet, it provides better results than the other approaches by giving a consistent annotation scored with a global criterion — instead of one score per concept.

## Background

Over the last decade, the data volume has been incessantly growing because of new or improved numerical technologies. In particular in biomedical domains, they provide anyone with the ability to create and share new contents — texts (e.g. scientific papers), pictures (e.g. radiographies, skin disease images), videos, etc. The management of massive collections is a problem that needs to be addressed by new methods capable of handling big datasets. One key process is document indexing [1], which associates each document with metadata so that the corpus can be more easily mined via applications such as information retrieval or recommending systems. This consists of assigning a document to one or several classes that can be represented by words. However, ambiguous words hamper such keyword-based applications [2]. Shall we use “tumor” or “carcinoma” in a query? Does “hospital” refer to the building or to the medical institution? There is also no relation considered between the words. “Neoplasms” and “carcinoma” are simply considered different in keyword-based applications whereas their meanings are pretty close. In order to overcome these problems, a widespread solution is to rely on knowledge representations such as ontologies [3,4]. The biomedicine field has devoted much effort to creating such structured vocabularies. Some examples are the Gene Ontology (GO), the Clinical Terms (SNOMED CT) or the Medical Subject Headings (MeSH). Annotations of entities (genes, biomedical papers, etc.) using such structured vocabularies are more informative since their concepts and the relations among them tackle the above-mentioned limitations of keyword-based approaches [5].

Indexing has been historically done manually (e.g. in libraries) but it tends to be automated due to the explosive growth in corpora sizes. Manual curation of data has been shown to be a very challenging and time-consuming task, particularly when annotations rely on an ontology, e.g. biomedical papers from MEDLINE^®;^ annotated with the MeSH [6]. Besides the number of documents to be processed, semantic indexing is a complex and subjective task and the indexers have to perfectly know and understand the underlying knowledge representation. The difficulty for a human expert to pick up the most accurate concepts to index a new document is better apprehended considering, for instance, that the 2014 edition of MeSH contains more than 27,000 descriptors/concepts.

Therefore, the annotation process is usually helped by algorithms in order to overcome time consumption problems. Those methods have been developped for several document media, e.g. for indexing images [7,8], texts [9], audio documents [10] or videos [11]. Indeed, irrespective of the media, authors agree that efficient annotation strategies are needed for real world data-driven applications. Hence, all of those indexing approaches aim to identify the most relevant concepts of a taxonomy to index a document. Thanks to technological advances in the early 2000’s, researchers have been able to automatically exploit knowledge representations. To the best of our knowledge, the biomedicine field was the first to propose algorithms and pipelines to automatically annotate biomedical papers with MTI (Medical Text Indexer) [12]. This project from the NLM’s (National Library of Medicine) Indexing Initiative proposed a two-fold strategy to annotate textual documents. First, concepts are both fetched and extracted. Second, this pool of concepts is ordered by relevance based on values computed during the first indexing phase to select each of those concepts.

The first phase of the indexing process is thus related to a retrieval task. Some of this retrieval is text-based and requires NLP (Natural Language Processing) tools. Many applications have been proposed to identify concepts in a text such as the NCBO annotator [13], MaxMatcher [14] and many more [15]. In MTI [12], extraction of UMLS concepts (Unified Medical Language System) is based on the title and abstract of a paper, using MetaMap [16], before being restricted to a set of MeSH concepts — with UMLS being a broader ontology. One other and common way to retrieve relevant concepts to annotate a document is to focus on already annotated documents that have similar contents. The assumption here is that similar documents should be similarly annotated. PMRA (PubMed Related Articles) [17] is an algorithm made to help users finding papers that are similar to a given one. Originally, this method estimates document similarity based on two criteria: text similarity — the words those documents have in common in their titles and abstracts — and semantic similarity — the relatedness of their MeSH annotations. This latter information is obviously not available during the indexing of a new document. Several papers such as [18-20] hence rely on a modified version of PMRA, that we further refer to as PMRA*, that finds similar documents by only using text similarity.

The second phase of the indexing process relies on information collected during the first one and on the related ontology to actually build up the index of the considered document. The work presented in this paper focuses on this second phase. In most existing methods this phase only orders the concepts collected during the first one. For instance, this ordering is done in MTI [12] based on concept relevance defined using criteria such as: the method used to include the concept in the annotation pool or the concept frequency (weight) in the document title/abstract and in the related documents. More recently, various Machine-Learning (ML) approaches were applied to learn the relevance of a concept for a given document: gradient boosting [21], reflective random indexing [22] and Learning-To-Rank (LTR) [18]. They all show better results than MTI. Yepes et al. [23] note that the effectiveness of ML methods is problem-dependent. They hence suggest that, instead of using one ML algorithm for annotating every document, it would probably be better to use meta-learning so that the system can choose the method to apply for a given document. ML thus appears to behave differently depending on the corpus and/or the ontology we use. However, the main limitation of ML methods is the compulsory learning phase: [23] reports 200,000 training citations for 100,000 tests, [22] trained the algorithm on more than 3,000,000 citations and [24] used 100,000 articles during the learning process. Considering the number of learning instances compared to the number of tests, the described ML methods are likely to overfit the training data.

Among all indexing models, Yang [25] and Trieschnigg et al. [26] both stated that the k-Nearest neighbors (k-NN) approach is the only method that can scale while providing good results. This approach is based on the neighborhood of the document to annotate. Each neighbor acts like a voter for each potential annotating concept. In basic applications, the most frequent concepts used in k-NN indexing are those proposed for annotating new documents. Huang et al. [18] present a more elaborate approach by mixing k-NN and ML methods, thus providing efficiency and effectiveness. First, the k-NNs of the document to be indexed are identified via the PMRA* — which is also used in MTI. The concepts indexing the k-NNs provide the set of concepts to start with. The concepts are then ordered using the LTR [27] algorithm that relies on a set of features, such as the concept frequency in the k-NN annotations and some text-specific features such as the word unigram/bigram overlap between a concept label and the title or abstract. Since the method just reorders the concepts, a cut-off is applied on the list so that only the top 25 concepts will be proposed. By combining k-NN and ML, their approach can scale to the whole MeSH domain and provide better results than a basic frequency-based k-NN method.

However, several limits of current semantic indexation methods motivated our work. Historically, a list of concepts ordered by relevance is proposed to the expert indexer. Since concept relevance scores are evaluated independently, the top concepts of this list may be very similar. In extreme cases, having a list of 25 variants of the same concept is obviously not the most helpful information for the expert indexer. Another limit is the need of training data, considering that the full texts are not always available [18]. Finally, previous studies barely mention the algorithm complexity or computation times, which are crucial to favor reactive systems and interactive processes.

Here we introduce a new method called USI for User-oriented Semantic Indexer. This method returns a concise, consistent and accurate conceptual annotation based on a global evaluation of the returned concept set. This method is said to be user-oriented because its output is a suggested annotation that can easily be checked by an expert rather than an ordered list of concepts in which the expert should pick the concepts that, put together, will provide a relevant and concise annotation. For example, if a system outputs the folowing concepts: 1. “Mammals”, 2. “Carnivora”, 3. “Dog”, 4.“Cat”; the expert is likely to keep only one of them. This will certainly be “Mammals” or “Carnivora” depending on what the text is focusing on. Our approach automatically prunes redundancy so this kind of situation should not happen and “Mammals” or “Carnivora” would be the only proposed concept. Moreover, a variant of USI relies on a semantic map to help the user to easily refine the set of k-NNs used to derive the annotation of the document to annotated. We also provide algorithm details that allow us to implement USI with a low time complexity and hence to propose a faster solution than that proposed by Huang et al. [18].

## Methods

In this section, we present step by step how USI differs from previous methods. Then, we detail the algorithm we used to implement it and how we optimized it to enhance its time performances. Our approach basically relies on semantic similarities to annotate a document with the medial annotation of its k-NNs.

### Identification of the k-NNs

**Figure 1:**
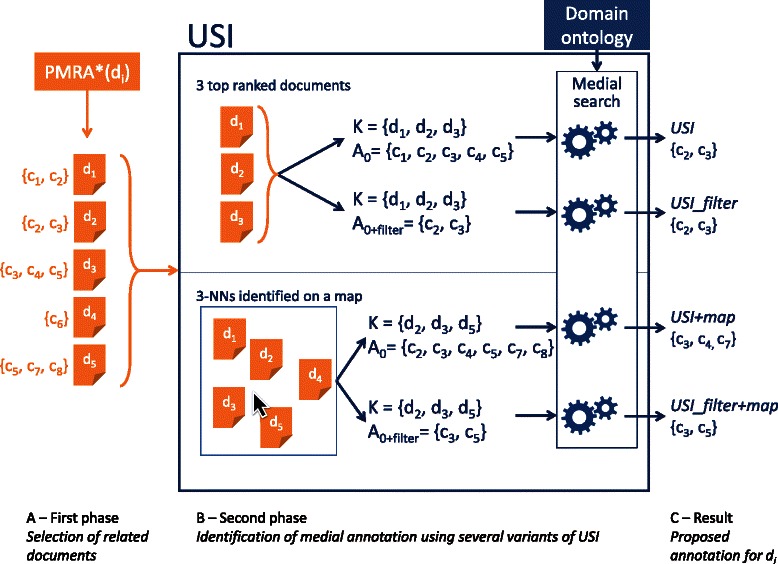
**Illustration of USI annotation process according to four variants.** During a first phase **(A)**, documents similar to the document *d*_*i*_ to be annotated are identified. Here, this is done using PMRA*. The set of the k-NNs of *d*_*i*_, denoted *K*, is identified from this ordered list. It will support the annotation calculus for *d*_*i*_. Here *k*=3. This selection may be done by only taking the *k* top ranked documents obtained by PMRA* (**B** top) or after interaction with the user on a semantic map (**B** bottom). The set *A*_0_ of candidate concepts for *d*_*i*_ annotation is obtained either by taking the union of concepts annotating at least one document (*A*_0_) or two documents (*A*_0+*filter*_) of *K*. This candidate set is then processed to find the medial annotation of *K* that will be proposed to annotate *d*_*i*_ **(C)**.

Considering a document *d*_*i*_^a^ to annotate, its *k* neighbors are selected among the ordered set of its related documents identified during the first annotation phase, e.g. using PMRA*. We tested two possible strategies to restrict this ordered list to *k* elements. In the first one the k-NNs are simply the top *k* documents of the ordered list returned by the first phase. In the second one the documents of this ordered list are displayed on a semantic map and the user is asked to click on the map region where *d*_*i*_ should be; the *k* documents that are the closest to this click are then used as k-NNs. Hereafter we denote by *K* the set of the k-NNs of *d*_*i*_. For example, Figure 1 depicts a small example where PMRA* returns 5 documents and USI relies on a neighborhood of 3 documents. The automatic selection of the 3 top documents (according to PMRA* score) leads to select *K*={*d*_1_,*d*_2_,*d*_3_} as 3-NNs for *d*_*i*_ whereas the click-based approach, involving the user interaction, leads to select *K*={*d*_2_,*d*_3_,*d*_5_} as 3-NNs for *d*_*i*_.

### Pairwise similarities beyond raw frequencies

Let *A*_*d*_ denote the annotation of a document *d*∈*K* and $\mathcal {A}_{K}$ the set of annotations of the *k* documents in *K*. In the example depicted in the top of Figure 1, *K*={*d*_1_,*d*_2_,*d*_3_} and $\mathcal {A}_{K} = \{\{c_{1}, c_{2}\}, \{c_{2}, c_{3}\}, \{c_{3}, c_{4}, c_{5}\}\}$.

In existing k-NN approaches, the relevance of a concept (w.r.t. *d*_*i*_ annotation) is mostly estimated based on its frequency in $\mathcal {A}_{K}$. This simple approach completely ignores concept similarities such that if a third of $\mathcal {A}_{K}$ annotations contains “dog” another third contains “cat” and the last third contains “mammals”, the “mammals” concept will only have a low score of 1/3 despite its presence (direct or through hyponyms) in all annotations of $\mathcal {A}_{K}$. We thus propose to use pairwise semantic similarity, taking advantage of a domain ontology, to assess the similarity between two concepts instead of the crude implicit binary measure (1= are identical, 0= are different) used when relying on concept frequency.

In USI, pairwise similarities are computed using the Semantic Measures Library [28] (SML) that proposes a large choice of pairwise measures. We choose to follow the method of Névéol et al. [29] who used the Lin [30] pairwise similarity. This measure is defined using the Information Content of a concept *c*, denoted *I* *C*(*c*). This IC, that measures the amount of information a concept provides, may be assessed thanks to a corpus analysis, as proposed by Resnik [31], or solely based on the concept position within the knowledge representation (ontology) [32]. We chose to use Seco’s IC [33], which does not take the corpora into account to keep the application generic. The Lin similarity relies on IC values of the two concepts to compare, say *c*_*x*_ and *c*_*y*_, and the IC value of their most informative common ancestor (MICA):

(1) $$ \pi_{Lin}(c_{x},c_{y}) = \frac{2*IC(MICA(c_{x},c_{y}))}{IC(c_{x})+IC(c_{y})}.  $$

### Groupwise similaritiy beyond the concept list

Let us now consider the example where all annotations of $\mathcal {A}_{K}$ contain “dogs”, “cat” and “mammals”. Obviously those three concepts are highly relevant for indexing *d*_*i*_ and they will be returned by existing k-NN approaches at the top of the suggested list of concepts. However, those three concepts are highly similar and hence redundant, there is probably no need to keep the three of them in the annotation $A_{d_{i}}$. USI overcomes this problem by scoring the whole set of proposed concepts using semantic groupwise similarities between a potential annotation of $A_{d_{i}}$ and the neighbor annotations $\mathcal {A}_{K}$. BMA (Best Match Average) [34], a composite average, is a well-balanced way to estimate those groupwise similarities. Denoted as *s* *i* *m*(*c*,*A*) the similarity between the concept *c* and the annotation *A* is defined as the maximum pairwise similarity between *c* and all concepts of *A*, i.e. $sim(c,A) = {max}_{c^{\prime } \in A} (\pi _{\textit {Lin}}(c,c^{\prime }))$. The BMA similarity between two annotations $A_{d_{i}}$ and $A_{d} \in \mathcal {A}_{K}$, denoted hereafter as $\phantom {\dot {i}\!}{sim}_{BMA}(A_{d_{i}},A_{d})$, is defined as:

(2) $$ \frac{1}{2|A_{d_{i}}|} \sum\limits_{c \in A_{d_{i}}}{sim(c,A_{d})} + \frac{1}{2|A_{d}|} \sum\limits_{c \in A_{d}}{sim(c,A_{d_{i}})}.  $$

In order to annotate a new document, we look for a concise annotation that is as similar as possible to those in $\mathcal {A}_{K}$. We thus search the annotation $A_{d_{i}}$ maximizing the objective function:

(3) $$ {\fontsize{9.8pt}{9.6pt}\selectfont{\begin{aligned} f\left(A_{d_{i}}\right) = \frac{1}{|\mathcal{A}_{K}|} \sum_{A_{d} \in \mathcal{A}_{K}}{{sim}_{BMA}\left(A_{d_{i}},A_{d}\right)} - \mu |A_{d_{i}}|. \end{aligned}}}  $$

The first part of $f(A_{d_{i}})$ tries to maximize the semantic similarity of the proposed annotation with those of the k-NNs whereas the second part favors a concise annotation by penalizing the annotation proportionally to its size. The *μ*∈[ 0,1] parameter controls the balance between those two objectives. It allows users to relax the threshold value on $f(A_{d_{i}})$ degradation when reducing the size of the annotation:

(4) $${} \left|\frac{1}{|\mathcal{A}_{K}|} \sum\limits_{A_{d} \in \mathcal{A}_{K}}{{sim}_{BMA}\left(A_{d_{i}},A_{d}\right) - {sim}_{BMA}\left(A_{d_{i}} \setminus \{c\},A_{d}\right)}\right|.  $$

### Hill-climbing heuristic

We define a set of concept *A*_0_ in which the solution will be searched. It is the set of concepts present in at least one annotation of k-NNs (see Figure 1 for some examples). Limiting the search to subsets of *A*_0_ instead of trying all possible concept sets of the ontology already tremendously reduces the processing time. One can wonder how useful it is to introduce new concepts in *A*_0_. That is, for example, adding the “mammals” concept in *A*_0_ if it already contains “cat”, “dog”, “rabbit”, “bat”, etc. Our tests showed that it complicates the computation while not significantly improving the output.

Even when reducing the initial pool of concepts with *A*_0_, however, a brute force approach is not tractable either since it would require us to evaluate $\phantom {\dot {i}\!}2^{|A_{0}|}$ candidate annotations. We thus rely on a greedy hill-climbing heuristic to provide accurate annotation with a very short computing time. A naive approach is, starting from *A*_0_, to test the removal of one concept and to actually remove it if this leads to an increase in $f(A_{d_{i}})$. The process stops when no concept can be removed without decreasing $\phantom {\dot {i}\!}f(A_{d_{i}})$. However, the result of this heuristic depends highly on the initial concept order and is often rapidly stuck at a local maximum. A more robust heuristic search consists of always testing the removal of each concept and removing the one that leads to the highest increase in $f(A_{d_{i}})$. As we will see, this heuristic provides accurate annotation with very short computing times thanks to the algorithmic optimizations introduced in the next section.

### Algorithmic optimizations

The evaluation of $f(A_{d_{i}})$ for a given annotation A requires computation of the BMA similarity between $A_{d_{i}}$ and all *A*_*d*_ annotations contained in $\mathcal {A}_{K}$. Since we only consider the annotation $A_{d_{i}} \subseteq A_{0}$ and by definition, $\forall A_{d} \in \mathcal {A}_{K},~A_{d} \subseteq A_{0}$, all those BMA similarities ultimately only depend on |*A*_0_|^2^ pairwise similarities that will be used numerous times during the heuristic. Pre-computing and saving those pairwise similarities in a matrix *M*_*ps*_ is thus the first obvious optimization. The exact complexity of this step depends on the pairwise similarity measure that is used. For Lin’s measure, assuming that ICs have been computed once and for all when loading the ontology with |*V*| vertices, *M*_*ps*_ can be initialized in $\mathcal {O}(|A_{0}|^{2} |V|)$ since in the worst case a concept has |*V*−1| ancestors among which the MICA is searched.

Once we have *M*_*ps*_, any $\phantom {\dot {i}\!}{sim}_{BMA}(A_{d_{i}},A_{d})$ can be computed by restricting *M*_*ps*_ to a submatrix $\phantom {\dot {i}\!}M_{ps}(A_{d_{i}},A_{d})$ preserving only columns corresponding to concepts of $A_{d_{i}}$ and rows corresponding to concepts of *A*_*d*_. $\phantom {\dot {i}\!}{sim}_{BMA}(A_{d_{i}},A_{d})$ then corresponds to half the average of the maximum values of $\phantom {\dot {i}\!}M_{ps}(A_{d_{i}},A_{d})$ rows plus half the average of the maximum values of $\phantom {\dot {i}\!}M_{ps}(A_{d_{i}},A_{d})$ columns. Finding those maximum values from scratch requires parsing of the whole matrix so that $\phantom {\dot {i}\!}{sim}_{BMA}(A_{d_{i}},A_{d})$ is computed in $\mathcal {O}(|A_{d_{i}}||A_{d}|)$. Where $S_{d_{max}}$ denotes the maximal size of an annotation of $\mathcal {A}_{K}$, testing the removal of a concept in a current annotation of *z* concepts requires computing $|\mathcal {A}_{K}|$ groupwise similarities, with each being computed in $\mathcal {O}({zS}_{d_{\textit {max}}})$. This test must be done *z* times to select the concept to be removed and *z* ranges from |*A*_0_| to 1 because concepts are removed one after another. Hence the overall time complexity of a naive implementation of our heuristic would be

(5) $$ \mathcal{O}\left(\sum\limits_{z=|A_{0}|}^{1}{|\mathcal{A}_{K}| z^{2} S_{d_{max}}}\right) = \mathcal{O}\left(|\mathcal{A}_{K}| |A_{0}|^{3} S_{d_{max}}\right).  $$

The key idea of the algorithmic optimization implemented in USI is that the BMA similarities needed to test the removal of a concept *c*_*r*_ from a candidate annotation $A_{d_{i}}$ can efficiently be derived by updating some values already computed to estimate $\phantom {\dot {i}\!}{sim}_{BMA}(A_{d_{i}},A_{d})$.

Given the submatrix $\phantom {\dot {i}\!}M_{ps}(A_{d_{i}},A_{d})$, let *c* *o* *l*(*c*) denote the column containing the similarities for the concept *c* of *A*. The maximum value in *c* *o* *l*(*c*) hence corresponds to *s* *i* *m*(*c*,*A*_*d*_). The sum of the maximum column values, hereafter denoted as *s* *u* *m* *M* *a* *x* *C* *o* *l* *s*(*M*_*ps*_), is thus equal to $\sum _{c \in A_{d_{i}}}{sim(c,A_{d})}$. Note that in order to update these values for the annotation *A*∖*c*_*r*_, it suffices to remove the maximum value observed in *c* *o* *l*(*c*_*r*_) in $\phantom {\dot {i}\!}M_{ps}(A_{d_{i}},A_{d})$ which is also the maximum value observed in *c* *o* *l*(*c*_*r*_) in *M*_*ps*_(*A*_0_,*A*_*d*_). Hence we pre-compute the maximum column values for the $|\mathcal {A}_{K}|$ sub-matrices *M*_*ps*_(*A*_0_,*A*_*d*_) with $A_{d} \in \mathcal {A}_{K}$. This is done once and for all in $\mathcal {O}\left (|\mathcal {A}_{K}||A_{0}|S_{d_{\textit {max}}}\right)$, the sumMaxCols values needed to test a concept removal are then obtained in constant times for each document. Therefore, the overall complexity for computing the *sumMaxCols* values is

(6) $${} {\fontsize{8.4pt}{9.6pt}\selectfont{\begin{aligned} \mathcal{O}\!\left(\!\!|\mathcal{A}_{K}||A_{0}|S_{d_{max}} \,+\, \sum\limits_{k=|A_{0}|}^{1}\!{k|\mathcal{A}_{K}|}\!\!\right) \!= \mathcal{O}\left(|\mathcal{A}_{K}||A_{0}|S_{d_{max}} + |A_{0}|^{2}|\mathcal{A}_{K}|\right)\!. \end{aligned}}}  $$

Similarly, the *s* *u* *m* *M* *a* *x* *R* *o* *w* *s*(*M*_*ps*_(*A*_0_,*A*_*d*_)) can be efficiently updated. This part is however a little trickier since the removal of *c*_*r*_ from the current annotation $A_{d_{i}}$ may change some *r* *o* *w*(*c*) maximum values if *c*_*r*_ is precisely the most similar concept with *c*. To efficiently update the maxRow values we hence have to keep, for each concept *c*_*r*_ of $A_{d_{i}}$, a list of the rows of *M*_*ps*_ for which the maximum values were precisely found using *c*_*r*_ and to keep for each concept *c* of *A*_0_ the list of *A*_*d*_ annotations containing *c*. The *s* *u* *m* *M* *a* *x* *R* *o* *w* *s*(*M*_*ps*_(*A*_0_,*A*_*d*_)) values and those two lists are initialized in $\mathcal {O}\left (|\mathcal {A}_{K}||A_{0}|S_{d_{\textit {max}}}\right)$; and the latter list needs no update thereafter. When we test the removal of a concept *c*_*r*_ we hence know exactly for which rows the maximum values need to be recalculated. For each updated *r* *o* *w*(*c*) we compute the difference between its new maximum value and its old one. This difference then just has to be added to the *s* *u* *m* *M* *a* *x* *R* *o* *w* *s*(*M*_*ps*_(*A*_0_,*A*_*d*_)) value of each annotation *A*_*d*_∋*c* to update them. When iteratively testing the removal of each concept *c*_*r*_ of $A_{d_{i}}$, the maximum value of each of the |*A*_0_| rows will be updated, in $\mathcal {O}(|A|)$, exactly once. It follows that each *s* *u* *m* *M* *a* *x* *R* *o* *w* *s*(*M*_*ps*_(*A*_0_,*A*_*d*_)) is updated exactly |*A*_*d*_| times. The complexity for computing those *sumMaxRows* is thus

(7) $$ \mathcal{O}\left(|\mathcal{A}_{K}| |A_{0}| S_{d_{max}} + \sum\limits_{k=|A_{0}|}^{1}{|A_{0}| k + |\mathcal{A}_{K}| S_{d_{max}}}\right)  $$

(8) $$ = \mathcal{O}\left(|\mathcal{A}_{K}| |A_{0}| S_{d_{max}} + |A_{0}|^{3}\right).  $$

Note that we only detail how to proceed when removing a concept *c*_*r*_. When a concept *c*_*r*_ is temporarily removed, all previous values are cached such that the restoration does not require their re-computation, which is done with the same time complexity as the removal. The whole complexity of our optimized algorithm is thus the complexity needed to compute the *sumMaxCols* and *sumMaxRows* values:

(9) $$ \mathcal{O}\left(|\mathcal{A}_{K}| |A_{0}| S_{d_{max}} + |A_{0}|^{3}\right).  $$

Let us now detail the space complexity of this algorithm. This space complexity depends on the solution used to parse and store the domain ontology, which could be influenced by the used pairwise similarity. Following the assumptions done in the time complexity section, we consider here the Lin’s measure and assume that the domain ontology IS_A structure is stored in memory as well as the IC of each of its concept. Considering an ontology of |*V*| concepts a memory space of $\mathcal {O}(V^{2})$ is required to store those ontology related information — since in the worst case an ontology with |*V*| concepts has $\mathcal {O}(V^{2})$ IS_A-relationships. Moreover, the space complexity of USI optimized hill-climbing algorithm is dominated by the storage of the *M*_*ps*_ matrix of size |*A*_0_|^2^. *sumMaxCols* and *sumMaxRows* both are stored in $\mathcal {O}(|A_{0}|)$, so the overall space complexity of USI is

(10) $$ \mathcal{O}\left(V^{2}+|A_{0}|^{2}+2|A_{0}|\right)=\mathcal{O}\left(V^{2}\right).  $$

In average, we observe a memory usage of 300−400MB when processing the evaluation dataset — note that this includes the storage of the domain ontology, all the ICs and the matrix *M*_*ps*_ of pairwise similarities.

### Evaluation

The most recent work about automatic indexing for which evaluation datasets were provided is the one used to validate the method of Huang et al. [18], which we further refer to as LTR (Learning-To-Rank, the technique they used for ranking the suggested concepts) as compared to MTI [12]. This dataset provides expert curated annotations for 1,000 documents as well as the list of their 50 nearest neighbors (k-NNs) gathered via the PMRA* algorithm. Each neighbor is characterized by a title, a proximity score (provided by PMRA*) an abstract and a list of MeSH terms but the two latter may be missing for some neighbors.

We test several variations of our algorithm that are schematically summarized on Figure 1. The most basic version denoted *USI* is exactly the heuristic described in the previous section with the subset of the 20 nearest neighbors, i.e. the exact same k-NNs as in the LTR evaluation. We also test a USI functionality, called *filter*, in which the initial set of concepts *A*_0_ is pre-filtered to keep only those appearing in at least two annotations of the 20 considered neighbors. The objective of this variant is twofold: i) to reduce the average computing times, ii) to reduce the potential noise (hence potential local optima) introduced by concepts that appear only once in the k-NNs and are thus probably too document specific to be relevant. The third version, denoted as *USI+map*, relies on user interaction with a semantic map to select the 20 best neighbors among the 50 related documents gathered by PMRA*. This semantic map is computed using multidimensional scaling to position the 50 PMRA* documents on a 2D map so that their pairwise similarities (*s* *i* *m*_*BMA*_) are respected as much as possible. The expert only needs to pinpoint the location where the document to be annotated should be on this map, to implicitly specify its 20 k-NNs (according to their map locations). In our tests, we assume the expert pinpoints the correct location (i.e. the location corresponding to the expert annotation used as reference in the benchmark). The fourth and final version, *USI_filter + map*, is a straightforward combination of the filter and the map. Previous approaches from the state of the art assign a score to any concept that can be relevant for annotating the new document. The concepts are then ordered considering their scores and a cut-off is applied to pick the top ones — e.g. 25 in Huang et al. [18]. Our method differs from these previous works by returning a set of concepts that taken as a whole, is supposed to provide an overall description of the document that satisfies the user. The heuristic we defined removes the concepts until the objective function score does not increase anymore. Therefore, USI does apply a cut-off but proposes a variable number of concepts. While cut-off-based approaches tend to maximize the recall, USI tries to maximize the precision by proposing the minimal set satisfying the objective function. We evaluate the results with three criteria. First, the *F-score*, which is a classical metric for assessing the quality of an annotation, and was used for evaluating LTR in [18]. Second, the semantic similarity between the proposed annotation and the expected one, as suggested by the analyses of Névéol et al. [29]. Here, this similarity is denoted *Semantic score* and it is measured using the average groupwise semantic similarity (Lin & BMA). Third, the *processing time*, which is also an important criterion to consider, since the main objective of such an approach is to ease (semi)-automatic indexing for large corpora and ontologies. Note that the PMRA* computing time is not taken into account for this criterion because (i) it has been used by all tested approaches so it should not impact the results and (ii) we used the PMRA* results given in the benchmark dataset and did not recalculate them.

## Results and discussion

**Figure 2:**
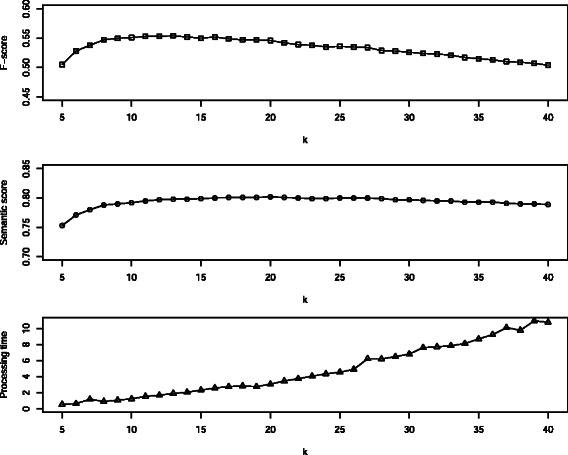
**Impact of the variation of** ***k*** **with the** PMRA* + USI **_** ***filter*** ***+ map*** **set-up.** Computing times are expressed in milliseconds. The highest values of the F-score and the semantic score are obtained with 10≤*k*≤20. Increasing the value of *k* over 20 would only increase the computation time while not providing better results.

We studied the impact of *k* on the output F-scores, semantic scores and processing time. The Figure 2 shows that the scores reach a plateau around *k*=10 that continues to *k*=20. The processing time, however, increases non-linearily while *k* increases. This leads to the same conclusion of previous work by Huang et al. [18] that *k* should be at most around 20. It also appears that an evaluation with a semantic measure is more robust than the F-score. The left part of the graphs shows low scores with both evaluations — because the output annotation is bad — but the right part diverges: the F-score decreases faster than the semantic score. This happens because as *k* increases, the concepts that are proposed are not exactly the ones of the gold standard but they are close to them. The F-score highly prunes such a substitution whereas the semantic score still considers the closeness between the gold standard and the substitution concept.

Table 1 summarizes the evaluations of MTI [12], the Huang et al. [18] LTR method, a naive implementation of the algorithm of USI detailed in Equation 5 and all set-ups of USI. Note that all USI scores are highly significantly higher (*p*<10^−6^) than previous approaches according to paired t-tests. The good semantic scores of MTI and LTR (resp. 0.68 and 0.768) compared to their low F-score (resp. 0.398 and 0.467) confirm the conclusions of Névéol et al. [29] that semantic scores better assess semantic annotation quality than F-scores. This is expected as the F-score similarly penalizes the complete absence of a concept and its replacement by a closely related concept. Those scores also confirm that LTR significantly outperformed MTI and proposed high quality annotations that are, on average, already very similar (0.768) to the expert ones.

**Table 1:** **F-score, semantic score and processing time for different methods with** ***k*** **=20**

**Method**	**F-score**	**Semantic score**	**Processing time (s)**
PMRA* + MetaMap + Clustering (MTI) [12]	0.398	0.68	N/A
PMRA* + LTR [18]	0.467	0.768	0.169
PMRA* + algorithm implemented in Equation 5	0.474	0.785	0.791
PMRA* + *USI*	0.474	0.785	0.015
PMRA* + *USI_filter*	0.521	0.776	0.003
PMRA* + *USI + map*	0.509	0.807	0.014
PMRA* + *USI_filter + map*	0.546	0.802	0.004

Note that PMRA* is never taken into account for the processing time since it has already been computed in the benchmark dataset. USI running times were measured on a 2.7Ghz microprocessor and 16Go of RAM Linux machine, whereas LTR running times, kindly provided by Huang et al., were measured on a somewhat comparable configuration but on a different machine.

The basic version of USI and its variant *USI_filter + map* both significantly outperforms LTR in terms of semantic-score (0.785 and 0.802, respectively, versus 0.768 for LTR) as well as in terms of the F-score (0.474 and 0.546, respectively, versus 0.467 for LTR). We also studied the specific benefits of the filter option and the use of the map. *USI_filter* mainly improves processing times (0.003 s versus 0.015 s for *USI* and 0.169 s for LTR) and the F-score as compared to USI alone. This happens because the filter reduces the search space *A*_0_ by removing the concepts annotating a single neighbor, thus reducing processing time and possible noise for the F-score. Remarkably, the combination of the filter option and the map in *USI_filter + map* allows their strengths to be combined, hence leading to a very high semantic score (0.802) and F-score (0.546) together with a low processing time (0.004 s).

All set-ups of USI are very fast and provide annotations in milliseconds. The difference with the processing time of the naive algorithm (0.791 s) proves that the optimization applied on USI highly improves its efficiency. Note however that those computation times are only indicative since it was not possible to run the LTR algorithm on the same machine as USI and its computation time is thus the one kindly provided by its authors but the low time complexity of USI is obviously an asset concerning this feature.

Although the map seems to improve the semantic score, we have to consider that, in a real context, the user would not instantly click on it. Therefore, the processing time should be higher when relying on the map. Since the scores do not greatly improve with the use of a map, we suggest using it only when the neighbors are highly heterogeneous. These cases are those with the worst scores but they are also those where using the map is the most useful to improve the suggested annotations. A natural extension of this work would thus be to detect whether or not the set of neighbors is heterogeneous — this can be assessed thanks to the initial *M*_*ps*_ matrix computed in USI. If it is, USI would propose a map to the user, otherwise a fully automatic method would be performed.

## Conclusions

In this paper, we present USI a fast and accurate solution for semantic annotation. By only using semantic annotations of the neighbor documents of the one to annotate, USI does not need any access to the abstract nor to the full text. USI annotations benefit from the definition of a global criterion of the proposed annotation based on semantic similarities. This global criterion enables USI to output consistent annotations — instead of individually scored ones — and allows it to outperform the approach of Huang et al. [18] based on their biomedical papers benchmark. USI is not only accurate, it is also fast thanks to the special care regarding algorithmic optimization leading to low computation complexity, thus ensuring that it can easily be scaled up to handle very large corpus and ontologies, even if larger neighborhood are considered to annotate a document. Another major advantage of USI, as compared to ML inspired strategies, is that no learning phase is needed, which avoids the problematic task of assembling a representative and accurate learning set. We are thus convinced that USI could be an efficient solution to the time consuming task of semantic annotation which is a pre-requisite for numerous semantic information management projects.

## Availability of supporting data

The data sets supporting the results of this article are available at http://kidknowledge.wp.mines-telecom.fr/software/usi.

## List of notations

*d*,*d*_*i*_ *:* one document (e.g. a scientific paper) *K* *:* a set of *k*=|*K*| identified neighbor documents $\phantom {\dot {i}\!}A_{d}, A_{d_{i}}$ *:* the annotation (set of concepts) of the document *d* and *d*_*i*_, respectively *A*_0_ *:* the set of concepts from which our method tries to find the best subset according to the objective function $\mathcal {A}_{K}$ *:* The set of annotations of documents in *K* i.e. $\mathcal {A}_{K}$ is a set of set of concepts *π*(*a*,*b*)*:* the pairwise similarity between two concepts *a* and *b* *π*_*Lin*_(*a*,*b*)*:* the Lin pairwise similarity between two concepts *a* and *b* [30] *I* *C*(*a*)*:* the information content, in this paper Seco’s IC, of a concept *a* [33] *M* *I* *C* *A*(*a*,*b*)*:* the most informative (with highest IC) common ancestor of concepts *a* and *b*. *c*,*c*_*x*_,*c*_*y*_ *:* concepts *s* *i* *m*(*a*,*B*)*:* the similarity between a concept *a* and a set of concepts *B* *s* *i* *m*_*BMA*_(*A*,*B*)*:* the Best Match Average similarity between two sets of concepts *A* and *B* *μ* *:* a parameter describing by how much the objective function score can decrease for the removal of one concept $f(A_{d_{i}})$ *:* the objective function *M*_*ps*_ *:* a matrix of pairwise similarities of concepts *V* *:* the vertices of an ontology (e.g. the Gene Ontology) : big O notation is used to define algorithms complexity, by assessing how they respond to changes in input size *M*_*ps*_(*A*,*B*)*:* the submatrix of *M*_*ps*_ with pairwise similarities of concepts from the sets *A* and *B* $S_{d_{\textit {max}}}$ *:* the maximal size of an annotation of $\mathcal {A}_{K}$ *c* *o* *l*(*a*)*:* the column of the matrix — or submatrix — corresponding to the concept *a* *r* *o* *w*(*a*)*:* the row of the matrix — or submatrix — corresponding to the concept *a* *c*_*r*_ *:* a concept tested for removal from *A*_0_ *s* *u* *m* *M* *a* *x* *C* *o* *l* *s*(*M*_*ps*_)*:* the sum of all column maximums in the matrix *M*_*ps*_ *s* *u* *m* *M* *a* *x* *R* *o* *w* *s*(*M*_*ps*_(*A*,*B*))*:* the sum of all row maximums in the submatrix *M*_*ps*_(*A*,*B*)

## Endnote

^a^ A comprehensive list of notations is provided at the end of this paper.

## Abbreviations

BMA: Best match average
GO: Gene ontology
IC: Informatic content
k-NNs: k-Nearest neighbors
LTR: Learning-to-rank
MeSH: Medical subject headings
ML: Machine-learning
MTI: Medical text indexer
NLM: National library of medicine
NLP: Natural language processing
PMRA: PubMed related articles
UMLS: Unified medical language system
USI: User-oriented semantic indexer
